# Celastrol induces cell cycle arrest by MicroRNA-21-mTOR-mediated inhibition p27 protein degradation in gastric cancer

**DOI:** 10.1186/s12935-015-0256-3

**Published:** 2015-10-24

**Authors:** Min Sha, Jun Ye, Zheng-yun Luan, Ting Guo, Bian Wang, Jun-xing Huang

**Affiliations:** Institute of Clinical Medicine, Taizhou People’s Hospital Affiliated of Nantong University of Medicine, Taizhou, 225300 China; Institute of Oncology, Taizhou People’s Hospital Affiliated of Nantong University of Medicine, 210 Yingchun, Taizhou, 225300 Jiangsu China

**Keywords:** Celastrol, Cell cycle arrest, miR-21, mTOR, p27

## Abstract

**Objective:**

Celastrol has anti-cancer effects by increase of apoptosis of gastric cancer cells. However, its role in gastric cancer cell cycle is still unclear. The aim of this study was to investigate the effect and mechanism of celastrol on gastric cancer cell cycle.

**Methods:**

The effects of celastrol on cell cycle in BGC-823 and MGC-803 cells were assayed via flow cytometry analysis. The expression of p27 and mTOR was detected by real-time PCR and western blot. The activity of mTOR and mTORC2 was measured by mTOR and mTORC2 kinase assays. miR-21 mimic was used to up-regulate miR-21 expression and mTOR expression plasmid was used to increase mTOR level in gastric cancer cells.

**Results:**

Celastrol caused G2/M cell-cycle arrest that was accompanied by the down-regulation of miR-21 expression. In particular, miR-21 overexpression reversed cell cycle arrest effects of celastrol. Further study showed that celastrol increased levels of the p27 protein by inhibiting its degradation. miR-21 and mTOR signaling pathway was involved in the increase of p27 protein expression in BGC-823 and MGC-803 cells treated with celastrol. Significantly, miR-21 overexpression restored the decrease of mTOR activity in cells exposed celastrol.

**Conclusions:**

The effect of celastrol on cell cycle arrest of gastric cancer cells was due to an increase of p27 protein level via inhibiting miR-21-mTOR signaling pathway.

## Background

There is increasing evidence demonstrating uncontrolled gastric cancer cell growth is due to disruption of the G1/S and G2/M checkpoints, which is involved in a series of positive and negative cell-cycle regulators [1, 2]. As a cyclin-dependent kinase inhibitor, p27 could negatively modulate cell cycle progression through inhibition of the G2/M phase [3]. Accumulating evidence suggests p27 was a useful predictive marker of prognosis of gastric cancer. The decrease of p27 expression facilitates tumor growth and correlates with clinical invasiveness of the tumors, while high expression of p27 was associated with a favorable prognosis [4, 5]. Therefore, it is important to make clear the key molecular mechanism of regulating p27 in gastric cancer.

As an oncogene, microRNA-21 (miR-21) plays an important role in regulating gastric cancer cell proliferation and migration [6]. High miR-21 expression predicts poorer survival in patients with gastric cancer [7]. Furthermore, overexpression of miR-21 is correlated with worse tumor differentiation, lymph node metastasis, and TNM stage [8]. When the expression of miR-21 was down-regulated in human gastric cancer, it was showed to inhibit cell proliferation [9].

The mammalian target of rapamycin (mTOR), a Ser/Thr kinase, plays essential roles in the regulation of growth-related processes [10]. It has been reported that the mTOR signaling pathway is activated in gastric cancer [11]. Activated mTOR has been positively correlated with tumor progression and poor survival in patients with gastric cancer [12, 13]. The mTOR inhibitor has been confirmed to induce cell cycle arrest and apoptosis of gastric cancer cell line [14]. Available clinical trial data show that the mTOR inhibitor is effective to treat gastric cancer patients in combination with chemotherapeutic agents [15].

Celastrol is a plant triterpene derived from the root of Thunder of God Vine and has anti-cancer effects [16]. Celastrol has also been found to induce apoptosis and autophagy of gastric cancer cells [17]. Our previous studies revealed that celastrol is capable of inducing apoptosis and inhibiting growth of gastric cancer cells through down-regulation of miR-21 expression [18]. However, whether celastrol exerts a negative effect role on gastric cancer cell cycle is still unclear. Therefore, in the present study, we investigated the effects and mechanisms of celastrol on cell cycle regulation. We hypothesized that celastrol induces cell cycle arrest of gastric cancer cells by modulating miR-21 expression and mTOR signaling pathway in BGC-823 and MGC-803 cells.

## Results

### Celastrol induced G2/M cycle arrest of gastric cancer cells

**Fig. 1:**
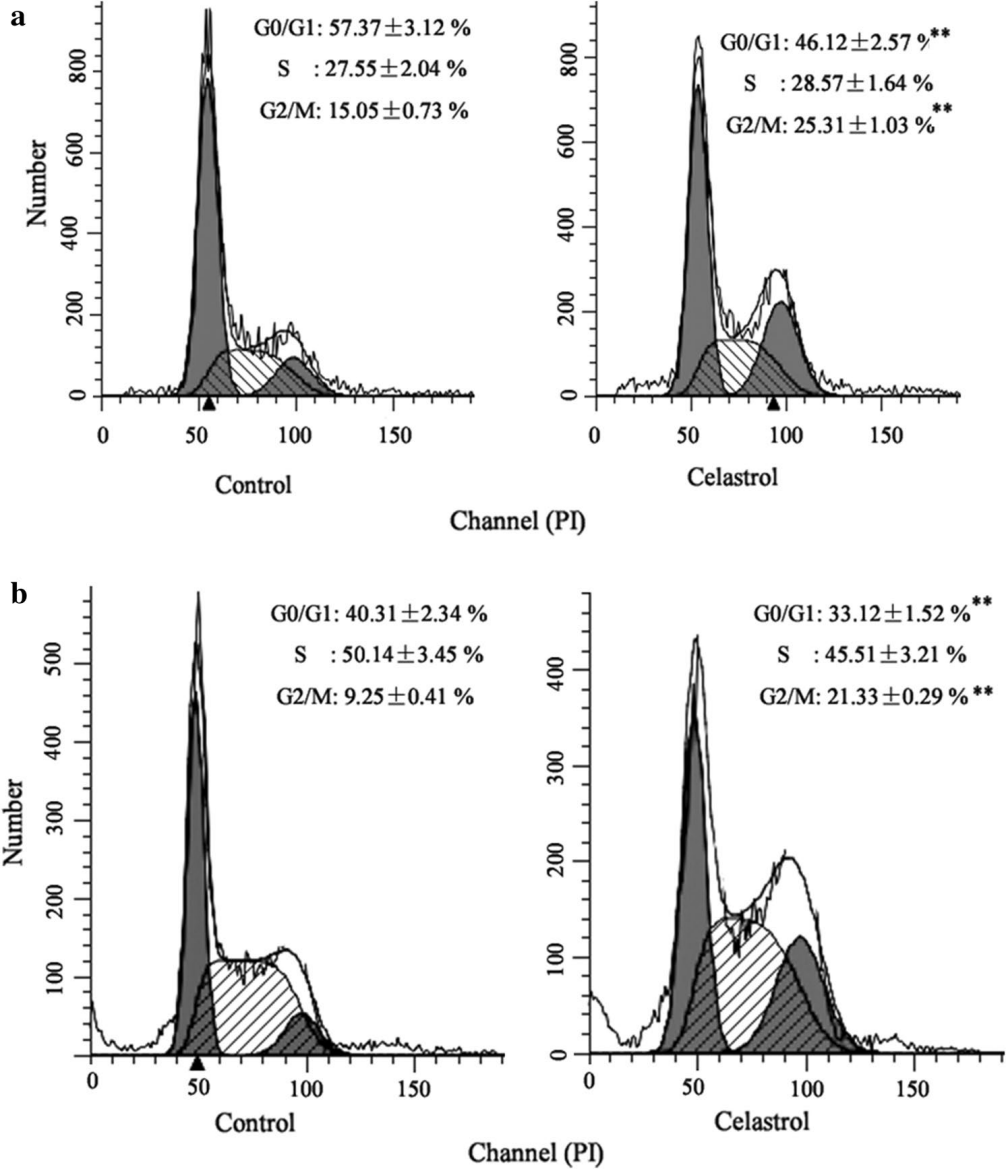
Celastrol induced G2/M cycle arrest of gastric cancer cells. Celastrol (2 μM) induced the accumulation of cells in the G2/M phase and decreased the cell population in the G0/G1 phases in BGC-823 (**a**) and MGC-803 cells (**b**). **P < *0.01* vs. control group

Our previous study showed that celastrol increased apoptosis of gastric cancer cells to exert anti-gastric cancer effect [19]. The growth of gastric cancer cells was significantly decreased after treated with celastrol for 24 h. To analyze the effect of celastrol on cell cycle regulation, we treated BGC-823 and MGC-803 cells with 2 μM celastrol and evaluated the cell distribution through the cell cycle. Celastrol induced the accumulation of cells in the G2/M phase and decreased the cell population in the G0/G1 phases (Fig. 1a, b).

Consistent to our previous study [18], we found that miR-21 expression was decreased in BGC-823 and MGC-803 cells treated with celastrol (data not shown).

### Up-regulation of miR-21 can reverse the effect of celastrol on cell cycle arrest

**Fig. 2:**
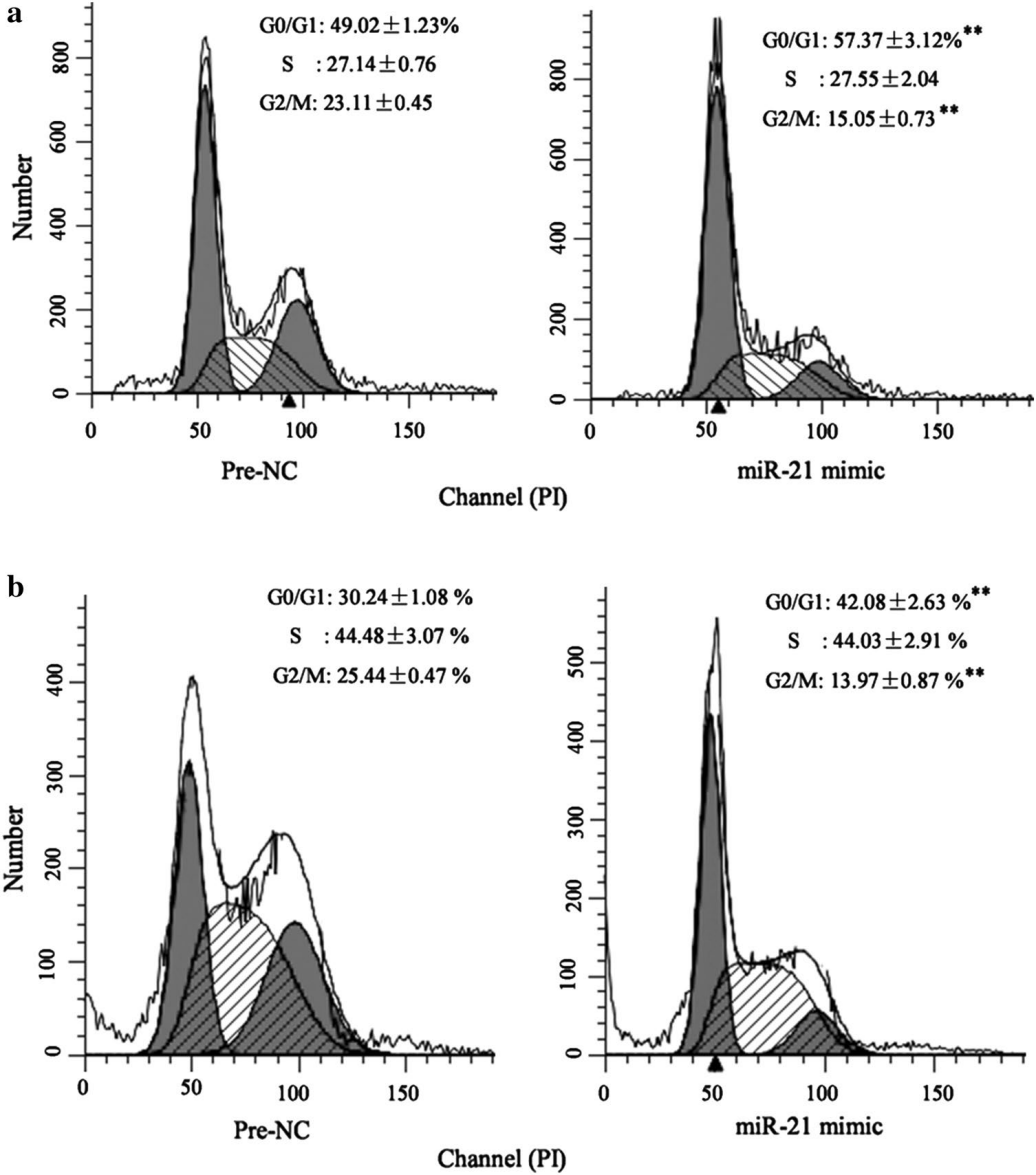
Up-regulation of miR-21 can reverse the effect of celastrol on cell cycle arrest. Flow cytometry analysis revealed that miR-146a mimic reversed the increase of G2/M-phase and the decrease of the G0/G1-phase induced by celastrol in BGC-823 (**a**) and MGC-803 cells (**b**). *P < *0.01* vs. Pre-NC

This assay was conducted to investigate whether miR-21 was involved in the effect of celastrol on cell cycle arrest in gastric cancer cells. In concordance with our prior findings, miR-21 mimic could reverse the increase of G2/M-phase and the decrease of the G0/G1-phase induced by celastrol in BGC-823 and MGC-803 cells (Fig. 2a, b).

### Celastrol increased p27 protein level in gastric cancer cells through miR-21

**Fig. 3:**
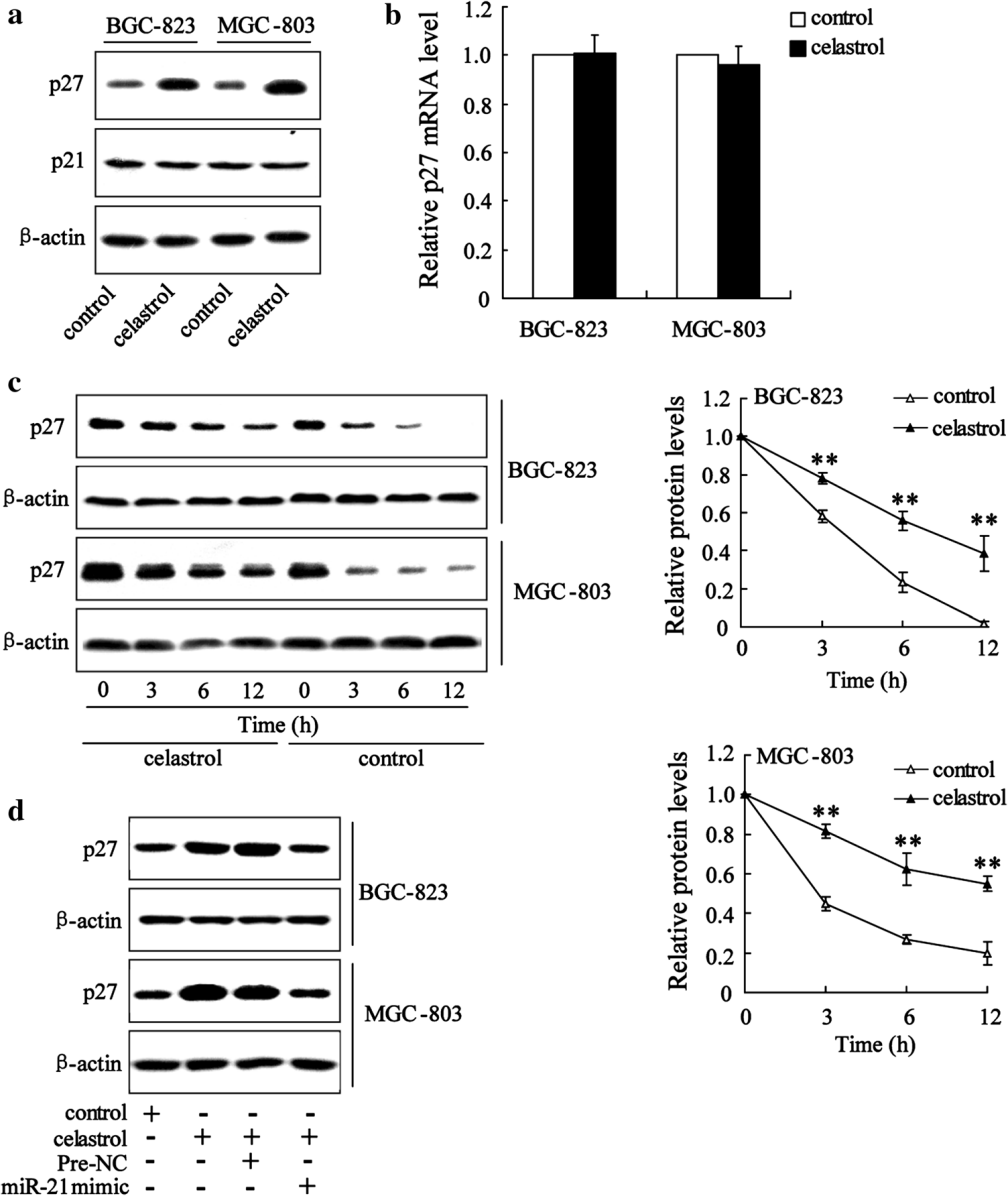
Celastrol increased p27 protein level in gastric cancer cells through miR-21. Western blot showed that celastrol significantly increased p27 protein level in BGC-823and MGC-803 cells (**a**). The real-time PCR revealed that celastrol had no effect on p27 mRNA level in BGC-823and MGC-803 cells (**b**). BGC-823and MGC-803 cells were treated without (control) or with celastrol for 24 h, at which time cycloheximide (50 μg/ml) was added for the indicated periods of time. All the treated cells were then harvested and lysed for western blot analyses to determine the protein levels of p27 and β-actin (as a loading control). Representative immunoblots and a graph showing protein levels of p27 relative to β-actin are shown (**c**). Western blot analyses revealed that miR-21 mimic reversed the increase of p27 protein level in BGC-823 and MGC-803 cells treated with celastrol (**d**). **P < *0.01*, indicate significant differences from the respective control groups

It has been reported that cell cycle regulators such as p21 and p27 have a particularly important role in anti-proliferative effect on gastric cancer cells [20]. Therefore, to further explore the mechanism involved in cell cycle arrest by celastrol in gastric cancer cells, the effect of celastrol on p21 and p27 expression was determined. As shown in Fig. 3a, b, celastrol increased the p27 protein levels but had no effect on P27 mRNA levels in BGC-823 and MGC-803 cells, indicating that celastrol upregulated the cellular level of p27 via a post-translational mechanism. To confirm this hypothesis, we measured the stability of the p27 protein after celastrol (2 μM) treatment using cycloheximide (50 μg/mL) to block de novo protein synthesis. As expected, the rate of p27 protein degradation was significantly inhibited in gastric cancer cells treated with celastrol (Fig. 3c). However, the p21 protein expression was not altered by celastrol (Fig. 3a).

Next, we explored whether miR-21 was implicated in p27 protein degradation induced by celastrol. The results showed that transfection of miR-21 mimic could decrease p27 protein level in gastric cancer cells exposed to celastrol (Fig. 3d).

### Celastrol inhibited mTOR activity through miR-21

**Fig. 4:**
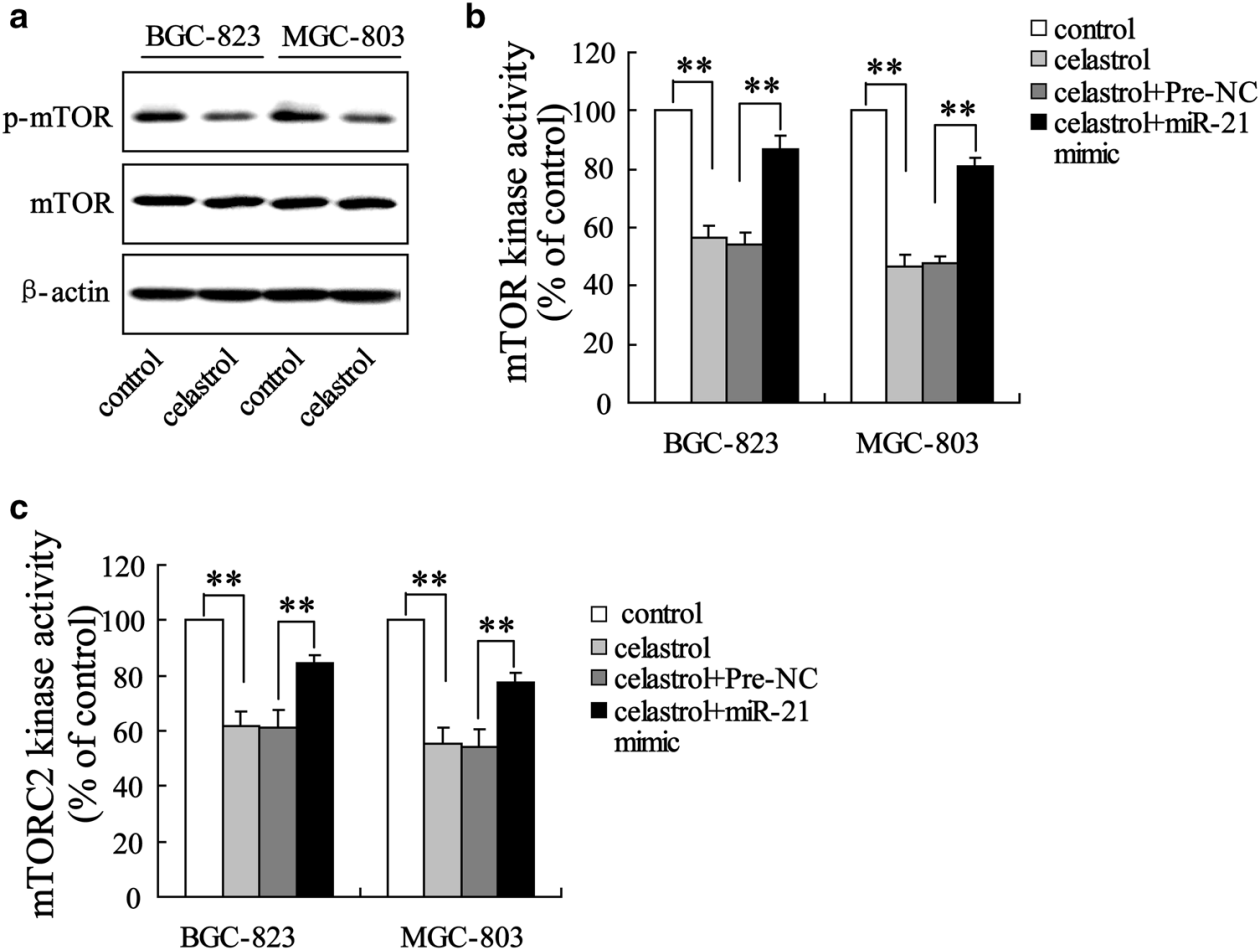
Celastrol inhibited mTOR activity through miR-21. Western blot showed that celastrol significantly decreased p-mTOR level in BGC-823and MGC-803 cells (**a**). mTOR and mTORC2 kinase assays revealed that miR-21 mimic reversed the decrease of mTOR (**b**) and mTORC2 (**c**) activity. **P < *0.01*, indicate significant differences from the respective control groups

Increasing evidences suggested that mTOR can be activated by Akt signaling pathway in gastric cancer [21, 22]. Because celastrol inhibited PI3 K/Akt activation in our previous study [18], we hypothesized that celastrol might interrupt mTOR activity in gastric cancer cells. Treatment of gastric cancer cells with celastrol significantly inhibited phosphorylation of mTOR, but did not affect total mTOR expression when compared with the control (Fig. 4a). In addition, we measured the mTOR activity by an mTOR kinase assay using recombinant protein. We observed that celastrol significantly decreased mTOR kinase activity, which could be reversed by miR-21 mimic (Fig. 4b). We also determined the effect of celastrol on mTORC2 immunoprecipitated from BGC-823 and MGC-803 cells lysates using a specific antibody against Rictor. The result also showed that mTORC2 kinase activity in BGC-823 and MGC-803 cells was significantly attenuated by celastrol administration (Fig. 4c). Similar to mTOR kinase activity, miR-21 mimic could restore the decrease of mTORC2 activity induced by celastrol.

### Down-regulation of miR-21 expression can inhibit mTOR activity

**Fig. 5:**
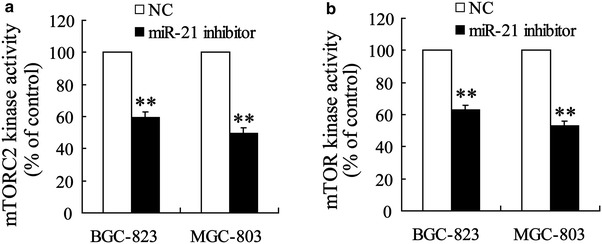
Down-regulation of miR-21 expression can inhibit mTOR activity. mTOR and mTORC2 kinase assays revealed that miR-21 inhibitor significantly reduced mTOR (**a**) and mTORC2 (**b**) activity. **P < *0.01*, indicate significant differences from the respective control groups

To determine whether miR-21 can regulate mTOR signaling pathway in gastric cancer cells, we investigated mTOR and mTORC2 kinase activity in cells transfected miR-21 inhibitor. The result showed that transfection of miR-21 inhibitor decreased mTOR and mTORC2 kinase activity as shown in Fig. 5a, b

### Celastrol increased p27 expression through mTOR

**Fig. 6:**
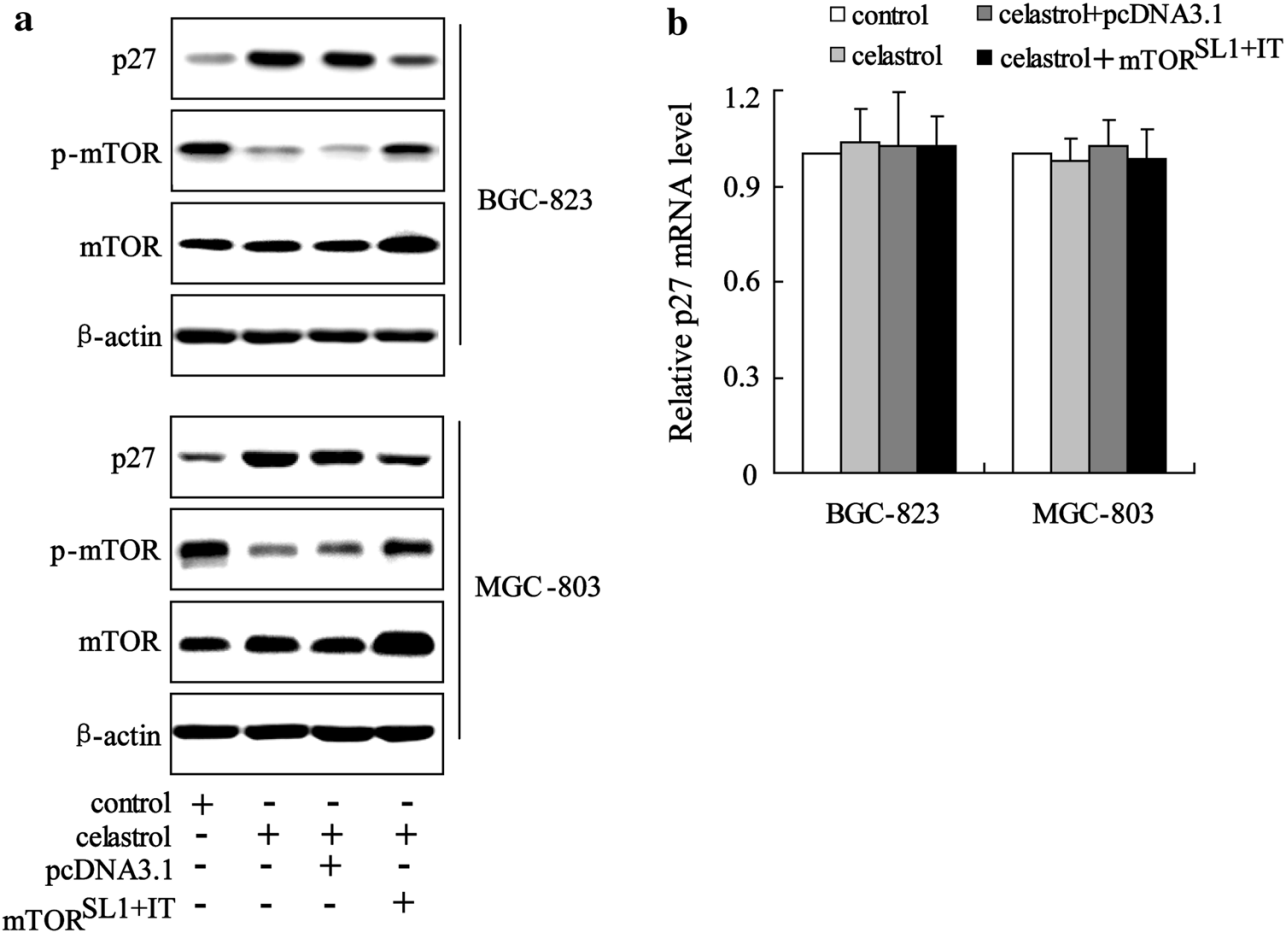
Celastrol increased p27 expression through mTOR. Western blot analyses revealed that mTOR^SL1+IT^ reversed the increase of p27 protein level in BGC-823 and MGC-803 cells treated with celastrol (**a**). The real-time PCR revealed that mTOR^SL1+IT^ had no effect of p27 mRNA level in BGC-823 and MGC-803 cells treated with celastrol (**b**)

In renal cell carcinoma cells, mTOR signaling increased the reduction of nuclear p27 protein levels [23]. Since celastrol significantly inhibited mTOR activity, we wanted to investigate whether mTOR played a role in celastrol regulating p27 protein degradation. Therefore, we transfected the mTOR^SL1+IT^ expression construct into BGC-823 and MGC-803 for 24 h, and then cultured the cells with or without celastrol for another 16 h. In gastric cancer cells treated with celastrol for 16 h, p27 was significantly reduced, while activation of mTOR rescued this protein level dramatically (Fig. 6a). Figure 6b shows that p27 mRNA level was not regulated by activation of mTOR and/or with celastrol treatment. These results suggested that inhibition of mTOR activity could maintain p27 protein levels.

## Discussion

The present report describes the molecular mechanism of cell cycle arrest induced by celastrol in human gastric cancer cells. Celastrol induced G2/M cycle arrest of BGC-823 and MGC-803 cells and caused the decrease of mTOR activity and the increase of p27 protein expression. Furthermore, up-regulation of miR-21 expression can reverse G2/M cycle arrest gastric cancer cells induced by celastrol. We also found that celastrol inhibited mTOR activity and enhanced p27 protein expression could be restored by up-regulation of miR-21 expression.

miR-21 is one of the most widely upregulated in a number of solid tumours, including gastric cancer [24]. The upregulation of miR-21 expression is positively related to shorter survival time and poorer prognosis in gastric cancer patients [25]. In gastric cancer cells, miR-21 not only inhibits apoptosis but also regulates cell cycle [8]. Thus, miR-21 is a therapeutic potential target for gastric cancer. Our previous study has demonstrated that celastrol can inhibit miR-21 expression and induce apoptosis of gastric cancer cells through miR-21 [18]. In the present study, our data showed that overexpression of miR-21 reversed a cell cycle arrest at G2/M phase induced by celastrol. These results suggest that celastrol induces cycle arrest of gastric cancer cell by inhibition of miR-21 expression.

We observed a strong upregulation of p27 but not p21 in celastrol-treated gastric cancer cells, indicating that promotion of p27 is important for celastrol regulation of cell cycle. Celastrol induced the increase of p27 protein levels but had no effect on p27 mRNA levels. In addition, the rate of p27 protein degradation was significantly decreased in BGC-823 and MGC-803 treated with celastrol. These results indicated that celastrol treatment inhibits p27 expression primarily through a post-translational mechanism. Overexpression of miR-21 almost completely prevented the effect of celastrol on p27 upregulation. According to these results, we draw a conclusion that celastrol increases p27 expression through miR-21.

Inhibition of the mTOR pathway suppressed the growth of gastric cancer in vitro and in vivo [26, 27]. In the present study, we demonstrated that celastrol markedly suppressed mTOR activity through inhibition of miR-21 expression. Moreover, the finding that down-regulation of miR-21 expression can inhibit mTOR activity provides further evidence that inactivation of mTOR is mediated by miR-21 in gastric cancer cells treated with celastrol. Inactivation of mTOR can promote p27 expression in various tumor tissues [28–30]. We also found that rapamycin, the inhibitor of mTOR, increased p27 expression in BGC-823 and MGC-803 cells (data not shown). Activation of mTOR rescued the increase of p27 protein level induced by celastrol (Fig. 6a). Therefore, we concluded that inhibition of mTOR contributes to the effects of celastrol on increased p27 expression.

## Conclusion

In this study, we established a primary relationship between celastrol and G2/M cycle arrest in BGC-823 and MGC-803 cells here. Furthermore, we explored celastrol-induced cycle arrest by miR-21-mTOR-p27 signaling pathways in gastric cancer cells. These results suggest that celastrol might be an effective drug to treat human gastric cancer.

## Methods

### Chemicals and reagents

Dulbecco’s modified Eagle’s medium (DMEM), sodium pyruvate were from Gibco-BRL (Rockville, MD, USA). Fetal bovine serum (FBS) was from PAA Laboratories (GmbH, Linz, Austria). Celastrol (purity > 98 %) was purchased from Alexis Biochemicals (San Diego, CA, USA). Leucine (purity 99 %) and dimethylthiazoly-2, 5-diphenyltetrazolium bromide (MTT) were obtained from Sigma Aldrich. Antibodies against mTOR, p-mTOR, p-S6, p-p70S6k, and p-BAD were purchased from Cell Signaling Technology (Beverly, MA, USA). Anti-Rictor, anti-p27 and anti-β-actin antibodies were purchased from Santa Cruz Biotechnologies (Santa Cruz, CA, USA). K-LISA™ mTOR activity kit was obtained from Millipore (Boston, MA, USA). The Detergent Compatible (DC) Protein Assay kit was purchased from Bio-Rad Laboratories (Hercules, CA, USA). The miRNeasy Mini kit, the miScript Reverse Transcription kit and the miScript SYBR Green PCR kit were purchased from Qiagen (Hilden, Germany).

### Cell culture

Human gastric adenocarcinoma cell line BGC-823 and gastric mucinous adenocarcinoma cell line MGC-803 were purchased from the Shanghai Institute of Cytobiology in the Chinese Academy of Sciences. BGC-823 and MGC-803 cells were cultured as described previously [18, 19]. All cell lines were maintained at 37 °C in a humidified incubator containing 5 % CO_2_ and treated with celastrol [dissolved in dimethyl sulphoxide (DMSO)] in complete DMEM medium. To obtain reliable results, the final concentration of DMSO in the culture medium was kept less than 0.1 %.

### Flow cytometry analysis

Cell cycle was analyzed by flow cytometry analysis. Before treatment, serum was deprived for 24 h to synchronize cell cycle. Then, serum was added back, and 2 μM of celastrol was added. After treatment with celastrol for 24 h, the cells were fixed with 80 % cooled ethanol, and incubated with 0.5 % Triton X-100 solution containing 1 mg ⁄ mL RNase A at 37 °C for 30 min. Next, propidium iodide (PI) was added into the wells at a final concentration of 50 μg ⁄ mL. Cellular DNA content was analyzed by a FACS (Becton Dickin-son). Data were processed using Cell-Quest software (Becton–Dickinson).

### Cell transfection

miR-21 was knocked down or overexpressed by transfection with miRNA inhibitor or miRNA mimic using siPort Neo-FX (Ambion) according to the manufacturer’s recommendations. The sequence of miR-21 mimics and miR-21 inhibitor were described as our previous report [18]. All of the oligonucleotides were synthesized by RIBOBIO (Ribobio Co. Ltd, Guangzhou, China) and transfected at a final concentration of 100 nM.

The expression plasmid for pcDNA3.1-FLAG-mTOR^SL1+IT^ plasmid (pYO293) was constructed as described previously [31]. Transient transfection was performed with LipofectAmine2000 (Invitrogen) according to the manufacturer’s instructions.

### Real-time PCR

Approximately 5 × 10^6^ cells were treated without (control) or with celastrol for 12 h. RNA was isolated and purified using Trizol reagent (Invitrogen, USA), according to manufacturer’s protocol. The p27 mRNA level was quantified by real-time PCR using TransStartTM SYBR Green qPCR Supermix (TransGen Biotech, Beijing, China), and with GAPDH as an internal normalized reference. For p27, the primers were as follows: forward, 5′-GTCAAACGTAAACAGCTCGAAT-3′ and reverse, 5′- TGCATAATGCTACATCCAACG-3′. For GAPDH, the primers were as follows: forward, 5′-TGGTGAAGGTCGGTGTGAAC-3′; and reverse, 5′-CCATGTAGTTG AGGTCAATGAAGG-3′.

### Western blot analysis

BGC-823 and MGC-803 cells were treated without (control) or with celastrol for 24 h. Then, cells were lysed with ice-cold lysis buffer containing: 50 mmol/l Tris–HCl, pH 7.4; 1 % NP-40; 150 mmol/l NaCl; 1 mmol/l EDTA; 1 mmol/l phenylmethylsulfonyl fluoride; and complete proteinase inhibitor mixture (one tablet per 10 ml; Roche Molecular Biochemicals, Indianapolis, IN, USA). Protein concentration in the cell lysate was quantified using the DC protein assay kit (Bio-Rad). After protein content determination using a DC Protein Assay kit, western blot analysis was performed. Rabbit anti-mTOR polyclonal antibody (pAb), rabbit anti-P-mTOR pAb and rabbit anti-p27 pAb were diluted 1:1000. Mouse anti-β-Actin monoclonal antibody was diluted 1:5000.

### mTOR and mTORC2 kinase assays

The in vitro mTOR kinase assay was measured using K-LISA™ mTOR activity kit, which utilizes a p70S6k GST fusion protein as a specific mTOR substrate. The assay was performed in accordance with instructions provided by the manufacturers. mTORC2 kinase assays using immunoprecipitated rictor complex proteins from BGC-823 and MGC-803 cells were performed as described previously. Briefly, mTORC2 kinase assays were carried out at 37 °C for 30 min in 25 mM Hepes (pH 7.4), 100 mM potassium acetate, 1 mM MgCl_2_, and 500 μM ATP, with rictor complex proteins as the substrate [32].

### Statistical analysis

Statistical analysis was performed with statistical analysis software SPSS 13.0 software. Statistical analyses were performed using either an analysis of variance (ANOVA) or Student’s *t* test. Data were expressed as mean ± standard deviation. *P* < 0.05 was considered to be significant.

## Abbreviations

miR-21: microRNA-21
DMEM: Dulbecco’s modified Eagle’s medium
FBS: fetal bovine serum
MTT: dimethylthiazoly-2, 5-diphenyltetrazolium bromide
DC: detergent compatible
DMSO: dimethyl sulphoxide
PI: propidium iodide
ANOVA: analysis of variance

## References

[1] Yang M, Zhong J, Zhao M, Wang J, Gu Y, Yuan X, Sang J, Huang C (2014). Overexpression of nuclear apoptosis-inducing factor 1 altered the proteomic profile of human gastric cancer cell MKN45 and induced cell cycle arrest at G1/S phase. PLoS One.

[2] Kim SJ, Lee HW, Baek JH, Cho YH, Kang HG, Jeong JS, Song J, Park HS, Chun KH: Activation of nuclear PTEN by inhibition of Notch signaling induces G2/M cell cycle arrest in gastric cancer. Oncogene. 2015 (Epub ahead of print). 10.1038/onc.2015.80 25823029

[3] Yuan CX, Zhou ZW, Yang YX, He ZX, Zhang X, Wang D, Yang T, Pan SY, Chen XW, Zhou SF (2015). Danusertib, a potent pan-Aurora kinase and ABL kinase inhibitor, induces cell cycle arrestand programmed cell death and inhibits epithelial to mesenchymal transition involving the PI3 K/Akt/mTOR-mediated signaling pathway in human gastric cancer AGS and NCI-N78 cells. Drug Des Devel Ther.

[4] Çalik M, Demirci E, Altun E, Çalik İ, Gündoğdu ÖB, Gürsan N, Gündoğdu B, Albayrak M (2015). Clinicopathological importance of Ki-67, p27, and p53 expression in gastric cancer. Turk J Med Sci.

[5] Aoyagi K, Kouhuji K, Miyagi M, Imaizumi T, Kizaki J, Isobe T, Shirouzu K (2013). Expression of p27Kip1 protein in gastric carcinoma. Hepatogastroenterology.

[6] Li L, Zhou L, Li Y, Lin S, Tomuleasa C (2014). MicroRNA-21 stimulates gastric cancer growth and invasion by inhibiting the tumor suppressor effects of programmed cell death protein 4 and phosphatase and tensin homolog. J BUON.

[7] Ma GJ, Gu RM, Zhu M, Wen X, Li JT, Zhang YY, Zhang XM, Chen SQ (2013). Plasma post-operative miR-21 expression in the prognosis of gastric cancers. Asian Pac J Cancer Prev.

[8] Wang Z, Cai Q, Jiang Z, Liu B, Zhu Z, Li C (2014). Prognostic role of microRNA-21 in gastric cancer: a meta-analysis. Med Sci Monit.

[9] Xu L, Dai WQ, Xu XF, Wang F, He L, Guo CY (2012). Effects of multiple-target anti-microRNA antisense oligodeoxyribonucleotides on proliferation and migration of gastric cancer cells. Asian Pac J Cancer Prev.

[10] Wang Z, Liu T, Chen Y, Zhang X, Liu M, Fu H, Liu D (2012). Inhibition of mammalian target of rapamycin signaling by CCI-779 (temsirolimus) induces growth inhibition and cell cycle arrest in Cashmere goat fetal fibroblasts (Capra hircus). DNA Cell Biol.

[11] Tapia O, Riquelme I, Leal P, Sandoval A, Aedo S, Weber H, Letelier P, Bellolio E, Villaseca M, Garcia P, Roa JC (2014). The PI3K/AKT/mTOR pathway is activated in gastric cancer with potential prognostic and predictive significance. Virchows Arch.

[12] Xiao L, Wang YC, Li WS, Du Y (2009). The role of mTOR and phospho-p70S6 K in pathogenesis and progression of gastriccarcinomas: an immunohistochemical study on tissue microarray. J Exp Clin Cancer Res.

[13] Wadhwa R, Song S, Lee JS, Yao Y, Wei Q, Ajani JA (2013). Gastric cancer-molecular and clinical dimensions. Nat Rev Clin Oncol.

[14] Fuereder T, Jaeger-Lansky A, Hoeflmayer D, Preusser M, Strommer S, Cejka D, Koehrer S, Crevenna R, Wacheck V (2010). mTOR inhibition by everolimus counteracts VEGF induction by sunitinib and improves anti-tumor activity against gastric cancer in vivo. Cancer Lett.

[15] Al-Batran SE, Ducreux M, Ohtsu A (2012). mTOR as a therapeutic target in patients with gastric cancer. Int J Cancer.

[16] Morita T (2010). Celastrol: a new therapeutic potential of traditional Chinese medicine. Am J Hypertens.

[17] Lee HW, Jang KS, Choi HJ, Jo A, Cheong JH, Chun KH (2014). Celastrol inhibits gastric cancer growth by induction of apoptosis and autophagy. BMB Rep.

[18] Sha M, Ye J, Zhang LX, Luan ZY, Chen YB, Huang JX (2014). Celastrol induces apoptosis of gastric cancer cells by miR-21 inhibiting PI3K/Akt-NF-κB signaling pathway. Pharmacology.

[19] Sha M, Ye J, Zhang LX, Luan ZY, Chen YB (2013). Celastrol induces apoptosis of gastric cancer cells by miR-146a inhibition of NF-κB activity. Cancer Cell Int.

[20] Wu WK, Cho CH, Lee CW, Fan D, Wu K, Yu J, Sung JJ (2010). Dysregulation of cellular signaling in gastric cancer. Cancer Lett.

[21] Wadhwa R, Song S, Lee JS, Yao Y, Wei Q, Ajani JA (2013). Gastric cancer-molecular and clinical dimensions. Nat Rev Clin Oncol.

[22] Chen H, Guan R, Lei Y, Chen J, Ge Q, Zhang X, Dou R, Chen H, Liu H, Qi X, Zhou X, Chen C (2015). Lymphangiogenesis in Gastric Cancer regulated through Akt/mTOR-VEGF-C/VEGF-D axis. BMC Cancer.

[23] Shanmugasundaram K, Block K, Nayak BK, Livi CB, Venkatachalam MA, Sudarshan S (2013). PI3K regulation of the SKP-2/p27 axis through mTORC2. Oncogene.

[24] Buscaglia LE, Li Y (2011). Apoptosis and the target genes of microRNA-21. Chin J Cancer.

[25] Wang Z, Cai Q, Jiang Z, Liu B, Zhu Z, Li C (2014). Prognostic role of microRNA-21 in gastric cancer: a meta-analysis. Med Sci Monit.

[26] Yu G, Fang W, Xia T, Chen Y, Gao Y, Jiao X, Huang S, Wang J, Li Z, Xie K (2015). Metformin potentiates rapamycin and cisplatin in gastric cancer in mice. Oncotarget.

[27] Han G, Gong H, Wang Y, Guo S, Liu K (2015). AMPK/mTOR-mediated inhibition of survivin partly contributes to metformin-induced apoptosis in human gastric cancer cell. Cancer Biol Ther.

[28] Hong F, Larrea MD, Doughty C, Kwiatkowski DJ, Squillace R, Slingerland JM (2008). mTOR-raptor binds and activates SGK1 to regulate p27 phosphorylation. Mol Cell.

[29] Ding XF, Yin DQ, Chen Q, Zhang HY, Zhou J, Chen G (2015). Validation of p27KIP1 expression levels as a candidate predictive biomarker of response torapalogs in patient-derived breast tumor xenografts. Tumour Biol.

[30] Zhao S, Lu N, Chai Y, Yu X (2015). Rapamycin inhibits tumor growth of human osteosarcomas. J BUON.

[31] Green AS, Chapuis N, Maciel TT, Willems L, Lambert M, Arnoult C, Boyer O, Bardet V, Park S, Foretz M, Viollet B, Ifrah N, Dreyfus F, Hermine O, Moura IC, Lacombe C, Mayeux P, Bouscary D, Tamburini J (2010). The LKB1/AMPK signaling pathway has tumor suppressor activity in acute myeloid leukemia through the repression of mTOR-dependent oncogenic mRNA translation. Blood.

[32] Lee E, Son JE, Byun S, Lee SJ, Kim YA, Liu K, Kim J, Lim SS, Park JH, Dong Z, Lee KW, Lee HJ (2013). CDK2 and mTOR are direct molecular targets of isoangustone A in the suppression of human prostate cancer cell growth. Toxicol Appl Pharmacol.

